# Enzymatic Transesterification of Kraft Lignin with Long Acyl Chains in Ionic Liquids

**DOI:** 10.3390/molecules200916334

**Published:** 2015-09-09

**Authors:** Lise Hulin, Eric Husson, Jean-Pierre Bonnet, Tatjana Stevanovic, Catherine Sarazin

**Affiliations:** 1 Unité de Génie Enzymatique et Cellulaire, FRE CNRS 3580, Université de Picardie Jules Verne, 33 Rue Saint-Leu, 80039 Amiens, France; E-Mails: lise.hulin@u-picardie.fr (L.H.); eric.husson@u-picardie.fr (E.H.); 2 Laboratoire de Réactivité et Chimie des Solides, UMR CNRS 7314, Université de Picardie Jules Verne, 33 Rue Saint-Leu, 80039 Amiens, France; E-Mail: jean-pierre.bonnet@u-picardie.fr; 3 Département des Sciences du Bois et de la Forêt, Centre de Recherche sur les Matériaux Renouvelables, Université Laval, 2425 Rue de la Terrasse, Québec, QC G1V 0A6, Canada; E-Mail: Tatjana.Stevanovic@sbf.ulaval.ca

**Keywords:** lignin, enzymatic esterification, lipase, ionic liquids

## Abstract

Valorization of lignin is essential for the economic viability of the biorefinery concept. For example, the enhancement of lignin hydrophobicity by chemical esterification is known to improve its miscibility in apolar polyolefin matrices, thereby helping the production of bio-based composites. To this end and due to its many reactive hydroxyl groups, lignin is a challenging macromolecular substrate for biocatalyzed esterification in non-conventional media. The present work describes for the first time the lipase-catalyzed transesterification of Kraft lignin in ionic liquids (ILs). Three lipases, three 1-butyl-3-methylimidazolium based ILs and ethyl oleate as long chain acyl donor were selected. Best results were obtained with a hydrophilic/hydrophobic binary IL system (1-butyl-3-methylimidazolium trifluoromethanesulfonate/1-butyl-3-methylimidazolium hexafluoro- phosphate, 1/1 *v*/*v*) and the immobilized lipase B from *Candida antarctica* (CALB) that afforded a promising transesterification yield (*ca.* 30%). Similar performances were achieved by using 1-butyl-3-methylimidazolium hexafluorophosphate as a coating agent for CALB rather than as a co-solvent in 1-butyl-3-methylimidazolium trifluoromethane-sulfonate thus limiting the use of hydrophobic IL. Structural characterization of lignin oleate was performed by spectroscopic studies (FTIR and ^1^H-NMR). The synthesized lignin oleate exhibited interesting thermal and textural properties, different from those of the original Kraft lignin.

## 1. Introduction

**Scheme 1:**
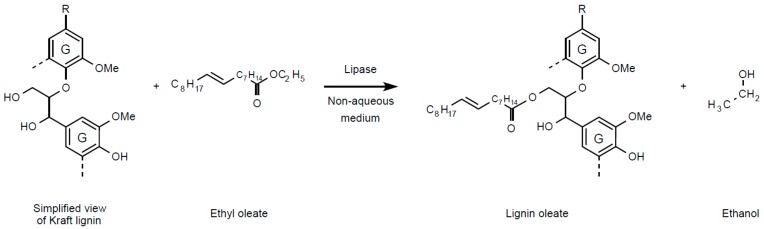
Reaction scheme of lipase-catalyzed transesterification of Kraft lignin with ethyl oleate as acyl donor in non-aqueous medium. Kraft lignin was simplified as guaiacyl unit (G) derivatives according to H/G/S ratio of 2/96/2 [37].

Valorization of industrial lignin, the second major polymer of lignocellulosic biomass (LCB) after cellulose, is essential for the economic viability of the biorefinery concept [1,2,3]. Lignins are complex highly branched amorphous polymers based on polyphenolic structure constituted of phenylpropane units, e.g., syringylpropane (S), guaiacylpropane (G) and hydroxyphenylpropane (H), providing interesting reactivity for chemical modification [4]. A current example of an industrial source of lignin is the paper industry where the alkaline pulping process generates around 50 millions of tons per year of Kraft lignin [5]. This by-product is largely undervalued and mainly used as black liquor constituent in the energy recovery cycle of the Kraft process. Other industrial applications are limited to low-added value products, mainly as base material for the production of chemicals, adhesives or fertilizers [6]. An emergent way of valorization could be the blending or reacting of Kraft lignin with apolar matrices of polyolefins to produce partially bio-based composites with improved rheological and thermomechanical properties and better carbon footprint [7,8,9,10]. However, the difference of polarity between Kraft lignins and polyolefins such as polyethylene impedes to a great extent their miscibility. To overcome this constraint, Kraft lignin hydrophobicity can be increased by chemical esterification with apolar moieties [11,12,13]. The acyl donors generally used for these modifications are the short acyl chains present in acetic, butyric, succinic or maleic anhydrides [8,11,14,15,16]. To our knowledge, only two patents describe chemical esterifications of lignin with long acyl chain compounds [17,18]. Drawbacks of chemical esterification can be the consumption of both an organic solvent such as dioxane and hazardous chemical reagents such as thionyl chloride (SOCl_2_). Other constraints are high reaction temperatures, extreme pH values and the production of by-products and salts [9]. On the other hand, biocatalysts such as lipases are commonly used in non-conventional (non-aqueous) solvents to catalyze ester bond synthesis on various biomolecules such as alcohols, amino-alcohols, peptides, flavonoids or cyclodextrins [19,20,21,22,23,24]. Advantages of the enzymatic pathway are the use of mild conditions, the specificity of reaction and a decrease in by-product production. Many constraints may govern the reaction outcome, such as substrate solubility, which could considerably limit its availability for enzyme reactivity/accessibility on the hydroxyl groups keeping in mind the need to maintain enzyme activity. To overcome these difficulties, a medium engineering approach coupled with a rational choice of biocatalysts have been found suitable for finding the appropriate solution allowing both substrate availability and high enzyme activity [25,26]. Ionic liquids (ILs) constitute interesting solvents with unique properties such as low vapor pressure, recyclability and the capacity to solubilize a wide range of biomolecules. ILs have been demonstrated to efficiently fractionate LCB under mild conditions in mediating selective extraction of lignin [27,28,29,30]. Some hydrophilic imidazolium-based ILs allow indeed the solubilization of hardwood and softwood Kraft lignins at moderate temperatures around 50–90 °C [31,32]. Likewise, some hydrophobic or water-miscible imidazolium-based ILs have been demonstrated to improve the activity of lipases and also the efficiency of enzymatic esterification of various alcohols [22,33,34]. To ensure consistence with biocatalysis requirements in minimizing toxicity risk, imidazolium-based ILs with cation alkyl chain lengths from 1 to 4 carbons have to be favored [35,36], such as those used in this work. In this present work, we describe for the first time the lipase-catalyzed transesterification of commercial Kraft lignin (Indulin AT) with ethyl oleate in a non-conventional medium (Scheme 1).

Ethyl oleate (C18:1) was chosen as long acyl chain donor for its known capacity to increase the hydrophobicity of polar biomolecules [20]. Three 1-butyl-3-methylimidazolium-based ILs differing in their constitutive anions (methylsulfate, trifluoromethanesulfonate and hexafluorophosphate) were selected for their ability to solubilize lignins or to maintain/improve lipase activity. 1,4-Dioxane was used as reference organic solvent. Three distinct immobilized lipase preparations: lipase B from *Candida antarctica* (CALB), lipase from *Pseudomonas cepacia* (PCL) and lipase of *Mucor miehei* (MML) were chosen for their capacity to catalyze esterification on aliphatic and phenolic hydroxyls [38,39,40]. The resulting modified lignin was then thoroughly characterized.

## 2. Results and Discussion

### 2.1. Lignin Solubility Study

The solubility of Kraft lignin was investigated in three 1-butyl-3-methylimidazolium-based ([C_4_C_1_im]) ionic liquids at 55 °C (Table 1). This temperature was selected based on previous works using lipases in non-conventional media [33,41]. [C_4_C_1_im][MeSO_4_], and [C_4_C_1_im][OTf] ILs are hydrophilic and lead to a high solubilization of lignin, about 29.3% and 14.7% (*w*/*v*), respectively. On the other hand, the hydrophobic [C_4_C_1_im][PF_6_] was demonstrated to be an unsuitable solvent to solubilize Kraft lignin with a very low solubility value of 0.03% (*w*/*v*). The solubilization of Kraft lignin in IL is dependent on its hydrogen-bond basicity, mainly influenced by the ILs’ constitutive anions [42,43,44]. Here, the Kamlet-Taft β parameter constitutes a pertinent indicator of the hydrogen-bond basicity of the solvent [45,46]. Solubility results show that lignin is efficiently solubilized in ILs with a high β parameter, probably due to strong coordination of its hydroxyl groups with the anion ([MeSO_4_] and [OTf]). On the contrary, non-coordinating anions such as [PF_6_] may not allow a good solvation of lignin as suggested in previous studies [28,32]. For comparison, 1,4-dioxane, usually used for enzymatic esterification of various biomolecules and chemical esterification of lignin [15,47,48] was tested. The solubility was significantly lower than those obtained in the two hydrophilic ILs (6.7% *w*/*v*). In conclusion, enhanced solubility can be achieved with hydrophilic ILs at mild temperatures. A Kraft lignin solution concentration of 1% *w*/*v* could thus provide a sufficient availability of substrate for enzymatic transesterification in hydrophilic ILs and dioxane. Although Kraft lignin was quite insoluble in the hydrophobic [C_4_C_1_im][PF_6_], the feasibility of enzymatic transesterification in this medium will be also investigated knowing the capacity to this IL to improve significantly lipase synthesis activity [20,22].

**Table 1:** Solubility of original Kraft lignin at 55 °C in the reaction media used for its enzymatic transesterification and their respective Kamlet-Taft β parameters.

Solvents	Solubility of Lignin % (*w*/*v*)	Kamlet-Taft β Parameter Value Reference	
Dioxane	6.7 ± 0.7	0.37	[46]
[C_4_C_1_im][MeSO_4_]	29.3 ± 1.3	0.60	[43]
[C_4_C_1_im][OTf]	14.7 ± 0.6	0.50	[44]
[C_4_C_1_im][PF_6_]	0.03 ± 0.01	0.21	[42]

### 2.2. Enzymatic Transesterification in Single ILs or Dioxane

**Figure 1:**
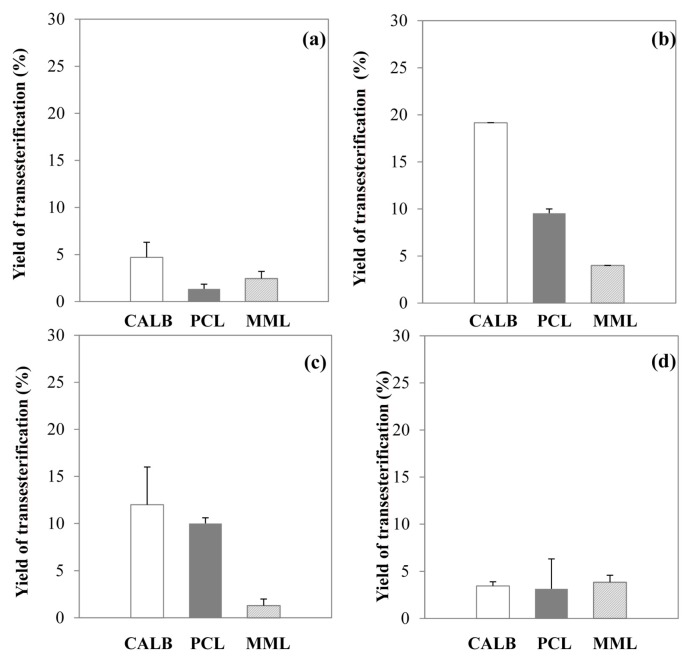
Yield of Kraft lignin transesterification with ethyl oleate (0.24 M) as acyl donor at 48 h catalyzed by the immobilized lipase B from *Candida antarctica* (CALB) in white bar, immobilized lipase from *Pseudomonas cepacia* (PCL) in grey bar and immobilized lipase from *Mucor miehei* (MML) in striped bar in dioxane (**a**); in [C_4_C_1_im][PF_6_] (**b**); in [C_4_C_1_im][MeSO_4_] (**c**); and in [C_4_C_1_im][OTf] (**d**) at 55 °C.

Figure 1a,b show that yields of transesterification in dioxane and [C_4_C_1_im][PF_6_] are below 5% whatever the lipase used. Thus, these media are not suitable for efficient enzymatic esterification of lignin. Although [C_4_C_1_im][PF_6_] is known to stabilize and improve enzymatic activity of lipases [33,34], the results highlight the importance of substrate solubilization in agreement with solubility values (Table 1). Significant improvement of transesterification yields in [C_4_C_1_im][MeSO_4_], can be observed (Figure 1c). CALB allows one to achieve an improved yield of 19.2% (*vs.* 4.7% in dioxane), and PCL 9.6% (*vs.* 1.4% in dioxane). Results with MML, are less significant with a low yield of 4% (*vs.* 2.5% in dioxane). The significant yield improvement obtained with CALB and PCL may result from a combination of good enzymatic activity and high Kraft lignin availability in this water-miscible IL. It could thus be suggested that the high hydrogen acceptor capacity due to the basicity of this IL may induce a strong solvation of lignin, following the deprotonation of its hydroxyl groups, as previously proposed in the literature for other biopolymers [49]. As a result, the ionized hydroxyl groups of lignin would become more nucleophilic allowing a more efficient attack on an acyl-enzyme complex. However, HPLC analysis has revealed the presence of an acyl by-product identified as methyl oleate up to a concentration representing 10% of initial concentration of ethyl oleate, for all lipases. As recently reported, the ester bond of the methylsulfate anion of [C_4_C_1_im][MeSO_4_] is hydrolytically unstable, leading to release of methanol CH_3_OH and hydrogen sulfate anion [HSO_4_^−^] [50]. Some factors in our reaction conditions such as temperature, residual water content of the lignin (3.07% ± 0.23% *w*/*w*) and acidic impurities in the IL, could induce this partial hydrolysis of [MeSO_4_] anion with a subsequent acid-catalysed methylation of oleyl chains. Due to this instability, [C_4_C_1_im][MeSO_4_] was not retained as a suitable reaction medium, although the yield of transesterification obtained with CALB is satisfactory.

[C_4_C_1_im][OTf] was then investigated as reaction medium, as its suitability for efficient enzymatic esterification of other compounds has been reported in the literature [51]. CALB and PCL lead to very close transesterification yields, 12% and 10%, respectively, whereas with MML a lower yield of 1.3% is obtained (Figure 1d). It can be hypothesized that MML would not be suitable to catalyze transesterification of Kraft lignin in this hydrophilic IL. The performance of CALB in this ILis less satisfactory than in [C_4_C_1_im][MeSO_4_]. It could be supposed that hydroxyl groups of lignin would be less nucleophilic in [C_4_C_1_im][OTf] than in [C_4_C_1_im][MeSO_4_] due to a weaker solvation effect according to the respective β parameters of these two ILs (0.50 *vs.* 0.60, respectively). Furthermore, it may be supposed here that [C_4_C_1_im][OTf] would induce a partial deactivation of CALB as already reported [51]. PCL may be less sensitive to the effect of enzymatic deactivation induced by [C_4_C_1_im][OTf] as illustrated by similar activity to that seen in [C_4_C_1_im][MeSO_4_]. No by-products have been detected in the [C_4_C_1_im][OTf] system. This hydrophilic IL, presenting therefore a higher stability against hydrolysis of the anion, allowed for significant enzymatic esterification of lignin, similarly to the hydrophobic IL usually used for enzymatic transformations of short chain alcohols [52].

### 2.3. Enzymatic Transesterification in Binary Hydrophilic—Hydrophobic IL Systems

**Figure 2:**
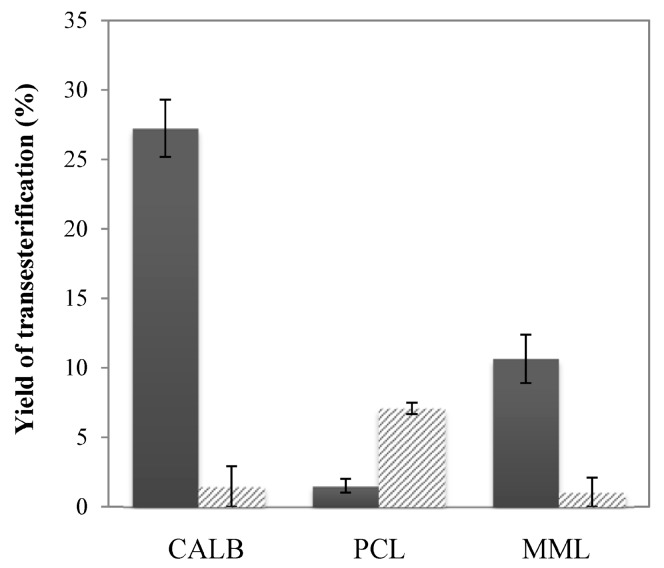
Yield of Kraft lignin transesterification with ethyl oleate (0.24 M) as acyl donor at 48 h catalyzed by the immobilized lipase B from *Candida antarctica* (CALB), the immobilized lipase from *Pseudomonas cepacia* (PCL) and the immobilized lipase from *Mucor miehei* (MML) in [C_4_C_1_mim][OTf]/[C_4_C_1_mim][PF_6_] (1/1, *v*/*v*) binary system at 55 °C: closed vials (grey bars) and open vials (striped bars).

To improve the efficiency of enzymatic transesterification, we first tested the combination of the hydrophilic IL [C_4_C_1_im][OTf] with the hydrophobic IL [C_4_C_1_im][PF_6_] as a binary system (1/1, *v*/*v*). Yields of Kraft lignin transesterification determined in this medium are presented in Figure 2 and show again a dependence on the immobilized lipase used. CALB led to the highest yield of 27.3% *vs.* 1.5% and 10.6% for PCL and MML, respectively. The very low yield with PCL suggests an important enzyme deactivation induced by the mixture of [C_4_C_1_im][PF_6_] and [C_4_C_1_im][OTf]. MML seems to be activated in the binary mixture contrary to the use of single ILs. In comparison to performances in the single ionic liquid [C_4_C_1_im][OTf], the binary system allows a significant increase the yield by a factor of 2.3 for CALB-catalysis and a factor of 8.1 for MML-catalysis, even higher than with the unstable [C_4_C_1_im][MeSO_4_] (Figure 1c). The efficiency of this enzymatic reaction system with CALB and MML is attributed to synergistic effects induced by the two ILs. On one hand [C_4_C_1_im][OTf] allows the efficient solvation of lignin by increasing the nucleophilicity of its hydroxyl groups. On the other hand, [C_4_C_1_im][PF_6_] preserves or improves the activity of some lipases [34,51].

In this work, transesterification was choose over esterification to avoid water production, however, accumulation of ethanol during the enzymatic transesterification with ethyl oleate in closed vials can be a limiting factor for the yield by displacement of the reaction equilibrium. In addition ethanol could induce inhibition of lipases by disturbing their surface hydration layer [53,54]. For these reasons, the syntheses in binary systems were also performed in open vials allowing partial ethanol evaporation from the reaction medium. The transesterification yields obtained at 48 h in open vials are presented in Figure 2. For the CALB and MML-catalyzed reactions, the performances decreased drastically in comparison with those obtained in closed vials. The yields lower than 2% could be explained by the atmospheric water trapped by the hygroscopic [C_4_C_1_im][OTf]. As a result, the hydrolytic activity of the two lipases was favored in agreement with the high concentrations of oleic acid detected at 48 h (0.16 ± 0.01 M in the reaction system with CALB and 0.09 ± 0.01 M with MML). Nevertheless, the transesterification yield obtained with PCL in open vials, although low, was increased by a factor of 4.6 in comparison with those obtained in closed vials. This improvement suggested that the immobilization support of PCL (Sol-Gel) may prevent water diffusion to the enzyme micro-environment, unfavorable to the hydrolytic activity (only 0.05 ± 0.01 M of oleic acid was detected in the reaction medium at 48 h). The implementation of enzymatic transesterification of lignin in binary systems in open vials was thus not considered further due to the hygroscopic properties of [C_4_C_1_im][OTf] favoring hydrolytic activity for the lipases rather than ester synthesis.

### 2.4. Enzymatic Transesterification in Hydrophilic IL with Hydrophobic IL-Coated Lipases

**Figure 3:**
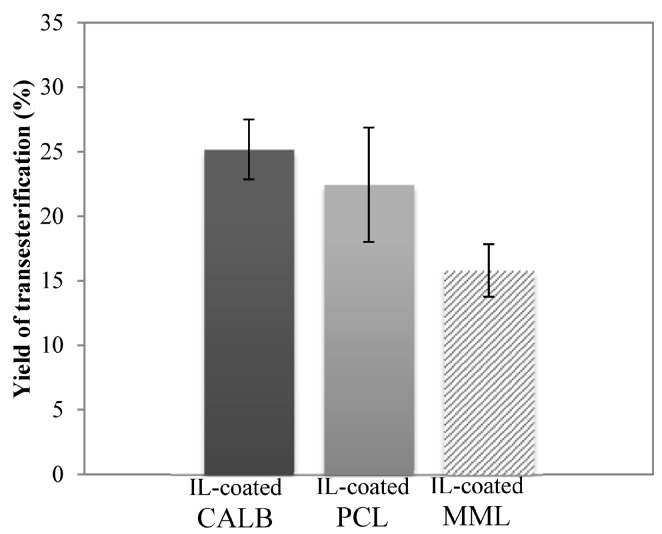
Yield of Kraft lignin transesterification with ethyl oleate (0.24 M) as acyl donor at 48 h catalyzed by the [C_4_C_1_mim][PF_6_]-coated immobilized lipase B from *Candida antarctica* (IL-coated CALB) in black bar, the [C_4_C_1_mim][PF_6_]-coated immobilized lipase from *Pseudomonas cepacia* (IL-coated PCL) in grey bar and the [C_4_C_1_mim][PF_6_]-coated immobilized lipase from *Mucor miehei* (IL-coated MML) in striped bar in [C_4_C_1_mim][OTf] as solvent at 55 °C.

To minimize the consumption of hydrophobic IL in our strategy, we proposed to test [C_4_C_1_im][PF_6_] as a coating for lipases instead of as a co-solvent. This approach has already shown to provide efficient catalytic performance and lipase stability [34]. Based on the results above, all experiments were performed in closed vials. Yields of Kraft lignin transesterification determined in this system are presented in Figure 3. 

CALB led to the highest yield of 25.2%, similar to those obtained in a binary system. The yield of transesterification achieved with [C_4_C_1_im][PF_6_]-coated MML was slightly improved in comparison with the binary system (15.8% *vs.* 10.7%). Interestingly, a significant increase in the yield was observed for [C_4_C_1_im][PF_6_]-coated PCL (22.4% *vs.* 1.5% in the binary system). According to literature data, the improvement obtained for these three enzyme preparations could be related to a better adsorption of [C_4_C_1_im][PF_6_] on their respective carriers leading to higher enzymatic activity [55,56].

### 2.5. Structural Characterization of Modified Lignin

**Figure 4:**
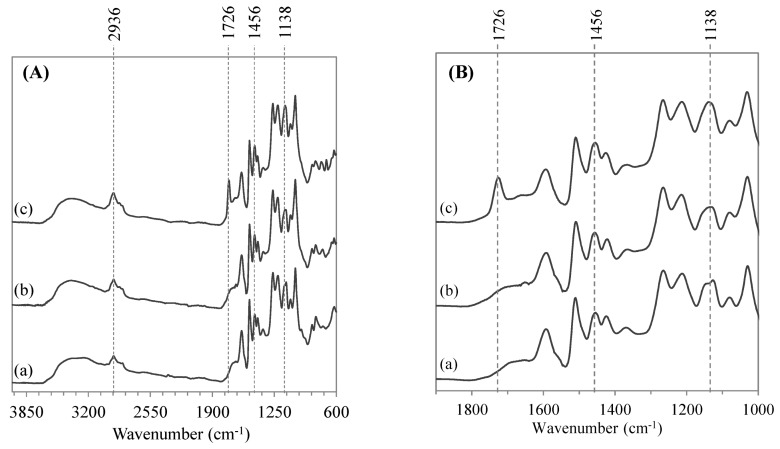
FTIR spectra (**A**) and their respective zooms of region from 1800 to 1000 cm^−1^ (**B**) of original Kraft lignin (a), control Kraft lignin extracted from the [C_4_C_1_mim][OTf]/[C_4_C_1_mim][PF_6_] binary system without enzyme (b) and lignin oleate extracted from [C_4_C_1_mim][OTf]/[C_4_C_1_mim][PF_6_] binary system after transesterification catalyzed by the immobilized lipase B from *Candida antarctica* (c).

Structural analyses are presented for enzymatically modified Kraft lignin extracted from the reaction system leading to the higher yield of transesterification, *i.e.*, [C_4_C_1_im][OTf]/[C_4_C_1_im][PF_6_] (1/1 *v*/*v*) binary system coupled with CALB. Figure 4 presents a FTIR spectrum of enzymatically modified Kraft lignin compared to spectra of original and control Kraft lignin (extracted from the binary system without enzyme), respectively.

Characteristic bands of ILs are totally absent from both control Kraft lignin and esterified lignin spectra, evidencing the efficiency of washing steps. Characteristic bands of original and control lignins were assigned in Table S1 (see Supplementary Information) and were consistent with literature data [37]. High similarity of the two FTIR spectra suggests that solubilization at low temperature (55 °C) in the [C_4_C_1_im][OTf]/[C_4_C_1_im][PF_6_] binary system without enzyme does not affect the molecular integrity of Kraft lignin. FTIR spectrum of enzymatically modified lignin displays a characteristic band at 1726 cm^−^^1^ assigned to the ester C=O that is a convincing proof of the enzymatic esterification of lignin [12,15,57]. In addition, a slight increase of C–H stretching band and C–H deformation bands at 2936–2875 cm^−1^ and 1456 cm^−1^, respectively, are attributed to the grafting of oleyl chains from the acyl donor. To a lesser extent, a decrease of the O-H phenolic and aliphatic bands can be observed at 3375 cm^−1^.With the appearance of a single band at 1726 cm^−1^ in the lignin oleate spectrum, it could be hypothesized that the aliphatic hydroxyls of lignin side chains would be preferentially esterified by CALB rather than phenolic hydroxyls [15].

**Figure 5:**
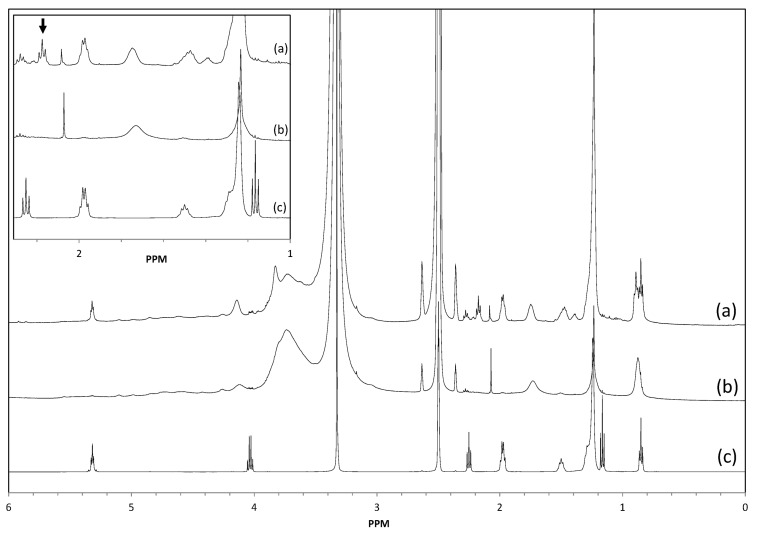
Respective ^1^H-NMR spectra of lignin oleate extracted from the [C_4_C_1_mim][OTf]/[C_4_C_1_mim][PF_6_] binary system with the immobilized lipase B from *Candida antarctica* (a); control Kraft lignin extracted from the [C_4_C_1_mim][OTf]/[C_4_C_1_mim][PF_6_] binary system without enzyme (b) and ethyl oleate (c).

Figure 5 presents the ^1^H-NMR spectrum of enzymatically modified Kraft lignin compared to spectra of control lignin and ethyl oleate, respectively. The modified Kraft lignin exhibits a characteristic proton triplet at 2.17 ppm assigned to the O(CO)CH_2_ methylene group from the oleyl chain ester (Figure 5a and insert). This signal is absent in the control lignin spectrum (Figure 5b and insert).

In ethyl oleate (Figure 5c and insert), this resonance is observed at 2.25 ppm. In previous studies on lignin acetate, the methyl signal of O(CO)CH_3_ was detected at 2.30 ppm for aromatic acetate protons and 2.15 ppm for aliphatic acetate protons. For lignin esters with longer acyl chains (propionate, butyrate, hexanoate), the O(CO)CH_2_ signals were observed at chemical shifts below 2.4 ppm for aliphatic ester protons [15,58]. Based on these results, an enzymatic oleylation of the lignin macromolecular structure mainly orientated towards its aliphatic hydroxyl groups was hypothesized. In addition, a signal at 5.31 ppm corresponding to the CH=CH function of the oleyl chain is present in the modified lignin spectrum and not in the control lignin spectrum. Moreover the resonances of the ethyl group of ethyl oleate are not present anymore in the enzymatically modified lignin (*i.e.*, the quadruplet at 4.03 ppm and the triplet at 1.16 ppm; Figure 5c).

### 2.6. Thermal Properties of Modified Lignin

**Figure 6:**
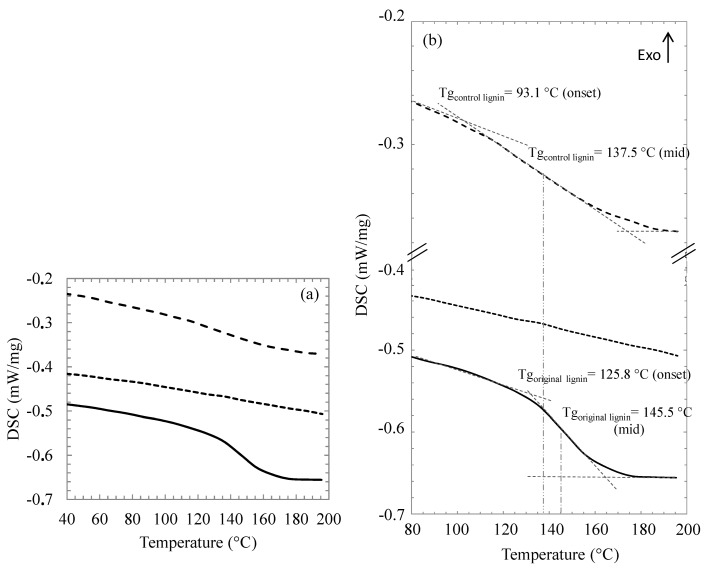
(**a**) Differential Scanning Calorimetry (DSC) thermograms of original Kraft lignin (continuous line); control Kraft lignin extracted from the [C_4_C_1_im][OTf]/[C_4_C_1_im][PF_6_] binary system without enzyme (large dotted line) and lignin oleate extracted from the [C_4_C_1_im][OTf]/[C_4_C_1_im][PF_6_] binary system after transesterification catalyzed by the immobilized lipase B from *Candida antarctica* (small dotted line) and (**b**) their respective zoomed versions.

Figure 6 presents the DSC thermograms for enzymatically modified lignin along with those of original and control Kraft lignins. 

For the original Kraft lignin a glass transition Tg at 125.8 °C (onset)/145.5 °C (mid) is clearly noticed, which corresponds to the literature data for Indulin AT [59]. Control Kraft lignin exhibited a Tg of 93.1 °C (onset)/137.5 °C (mid) which is lower but more difficult to determine than for original Kraft lignin (Figure 6b). This could suggest that Kraft lignin incubation in ionic liquid in the presence of an acyl donor without enzyme also affects its Tg. For lignin oleate, it was not possible to determine Tg in the range between room temperature and 200 °C. Previous studies showed that Tg of lignin esters synthesised by chemical routes decreased as the number of carbon atoms in the acyl chain increased [58,60,61,62,63]. For instance a Tg equal to 107 °C for an organosolv lignin decreases indeed to 2 °C for lignin laurate (12 carbons) [62]. Therefore, a Tg value below room temperature could be anticipated for lignin esterified with a long acyl chain of 18 carbons.

**Figure 7:**
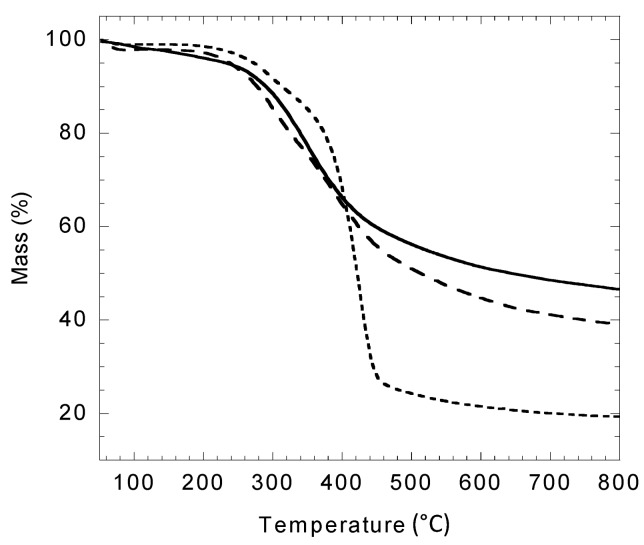
TGA analysis of original Kraft lignin (continuous line), control Kraft lignin extracted from the [C_4_C_1_mim][OTf]/[C_4_C_1_mim][PF_6_] binary system without enzyme (large dotted line) and lignin oleate extracted from [C_4_C_1_mim][OTf]/[C_4_C_1_mim][PF_6_] binary system after transesterification catalyzed by the immobilized lipase B from *Candida antarctica* (small dotted line).

The respective TGA thermograms of original Kraft lignin, control Kraft lignin and lignin oleate are shown on Figure 7. The first weak mass losses (a few percent) observed for all cases below 110 °C correspond to water evaporation from the samples (confirmed by mass spectrometry, not shown here). Then, large second mass losses, observed for all samples, are attributed to the decomposition of the macromolecular structure. This degradation starts at 276.8 °C (onset temperature, To) for the original Kraft lignin sample with a residual mass of 46.65% at 798 °C typical of the thermal decomposition of Indulin AT [37]. For control Kraft lignin the decomposition starts at To = 237.7 °C with a residual mass of 39.14% at 798 °C. For lignin oleate, a two-step degradation process occurs, with a first onset temperature at To_1_ = 251.4 °C followed by a second one at To_2_ = 372.4 °C and with a residual mass of 19.38% at 798 °C. The mass loss at 798 °C is significantly more important for lignin oleate compared to control and original Kraft lignins with a difference of 27.27% and 19.76%, respectively. This could be due to the presence of oleyl chains covalently grafted to lignin by enzymatic transesterification.

**Figure 8:**
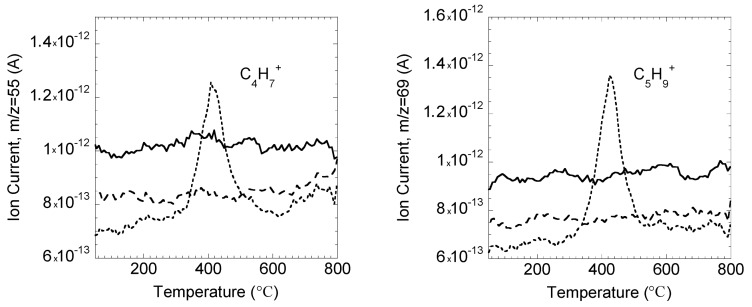
TGA-MS coupled analysis of original Kraft lignin (continuous line), control Kraft lignin extracted from the [C_4_C_1_mim][OTf]/[C_4_C_1_mim][PF_6_] binary system without enzyme (large dotted line) and lignin oleate extracted from the [C_4_C_1_im][OTf]/[C_4_C_1_im][PF_6_] binary system after transesterification catalyzed by the immobilized lipase B from *Candida antarctica* (small dotted line) as function of temperature.

In addition, the TGA analyzer was coupled with a low resolution mass spectrometer. Ion current intensities corresponding to two key ions, [C_4_H_7_]^+^ (*m*/*z* = 55) and [C_5_H_9_]^+^ (*m*/*z* = 69) as a function of temperature are presented in Figure 8. These ions are attributable to oleyl chain decomposition [64]. They are only noticeable for lignin oleate at about 410/430 °C. These TGA-MS analyses prove therefore undoubtedly the presence of oleyl chains in the case of enzymatically esterified Kraft lignin. The complete absence of these two ions in the original Kraft lignin and especially in the control Kraft lignin spectra constitutes direct proof of the absence of any residual adsorption of the acyl donor in lignin after extraction and washing steps. 

### 2.7. Textural Properties of Modified Lignin

**Figure 9:**
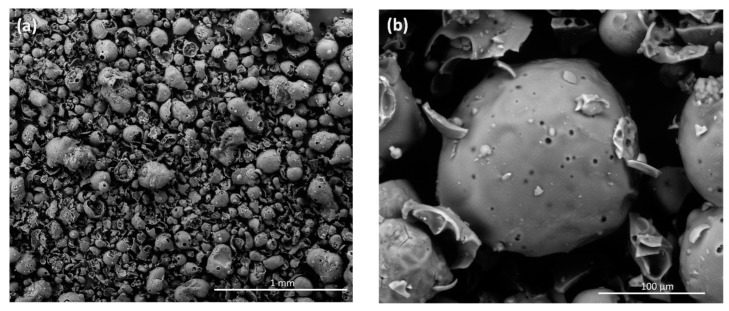

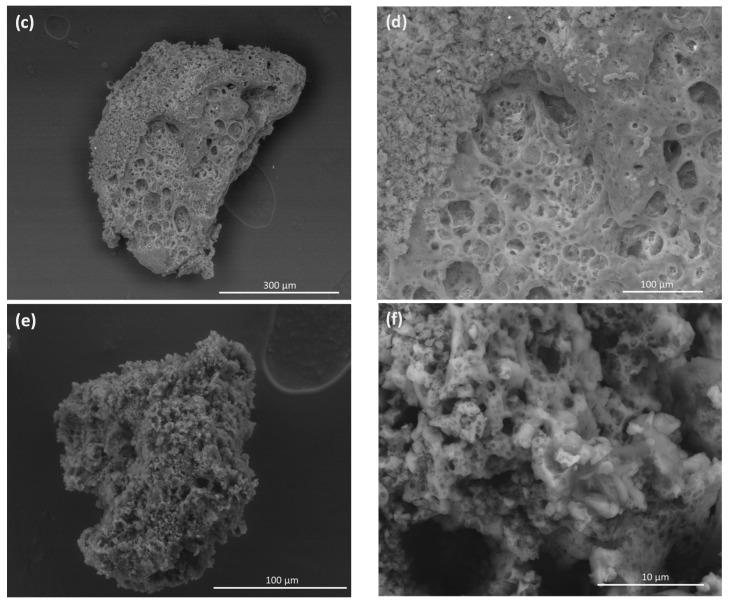
SEM micrographs of original Kraft lignin and their respective zooms (**a**,**b**); control Kraft lignin extracted from the [C_4_C_1_mim][OTf]/[C_4_C_1_mim][PF_6_] binary system without enzyme (**c**,**d**) and lignin oleate extracted from [C_4_C_1_mim][OTf]/[C_4_C_1_mim][PF_6_] binary system after transesterification catalyzed by the immobilized lipase B from *Candida antarctica* (**e**,**f**).

The morphology of original Kraft lignin, control Kraft lignin and lignin oleate has been investigated by scanning electron microscopy (SEM). Figure 9a presents a SEM micrograph of original Kraft lignin showing individualized rounded or (half)-spherical particles and also broken ones with heterogeneous sizes inferior to 200 µm. At greater magnification (Figure 9b), it can be observed that these particles are hollow and exhibit the smooth surfaces typical of Kraft lignin [65,66]. After extraction from the reaction medium without enzyme, control Kraft lignin completely lost its organization in spherical particles with a more compact morphology (Figure 9c). Heterogeneous microporosities between 5 and 40 µm are observed at higher magnification (Figure 9d). These structural transformations would be due essentially to the solubilization of lignin in the ionic liquid followed by its extraction with water. Significant structural changes are observed for lignin oleate in comparison with original and control Kraft lignins (Figure 9e). Indeed, lignin oleate has also lost its spherical particle organization and consists of a compacted material with a more homogeneous microporosity detectable at higher magnification (Figure 9f). These structural changes could be assigned to enzymatic transesterification with long acyl chains.

## 3. Experimental Section

### 3.1. Materials

A commercial Kraft lignin (Indulin AT) was received from Westvaco Corporation (New York, NY, USA). Ethyl oleate (98%), oleic acid (≥99%) and methyl oleate (≥99%) were acquired from Sigma-Aldrich (Steinheim, Germany). Dioxane with HPLC grade purity was from Fisher Scientific (Ilkirsch, France). 1-butyl-3-methylimidazolium methylsulfate [C_4_C_1_im][MeSO_4_] (98%), 1-butyl-3-methylimidazolium trifluoromethanesulfonate [C_4_C_1_im][OTf] (99.5%) and 1-butyl-3-methylimidazolium hexafluorophosphate [C_4_C_1_im][PF_6_] (99.9%) were produced by Solvionic SA (Verniole, France). Immobilized lipases—lipase B from *Candida antarctica* immobilized on acrylic resin (5000 PLU·g^−1^), lipase from *Pseudomonas cepacia* immobilized in Sol-Gel-AK (186 U·g^−1^) and lipase from *Mucor miehei* immobilized on a macroporous ion-exchange resin (140 U·g^−1^)—were furnished by Sigma-Aldrich.

### 3.2. Solubility of Lignin

The determination of Kraft lignin solubility was adapted from previous studies [27,28]. Dioxane or ILs (500 µL) were added into Pyrex tubes then progressively saturated with lignin at 55 °C under vigorous magnetic stirring for 24 h. After centrifugation (14,000 rpm, 10 min, 40 °C with an Allegra 64R Beckman Coulter Rotor: F (Beckman Roissy, France), the supernatant was recovered then diluted in 0.1 N NaOH aqueous solution. Measurements of total solubilized lignin in supernatant were performed by UV-Vis spectrophotometry (Cary 50 BIO, Varian, Les Ulis, France) at 280 nm based on calibration curves established using standard lignin (Indulin AT) solubilized in 0.1 N NaOH aqueous solutions. Each experiment was repeated in duplicate and solubility values were expressed as mean values with standard deviation (±) in % *w*/*v* (Table 1).

### 3.3. Enzymatic Esterification of Kraft Lignin

Enzymatic esterification reactions of Kraft lignin in dioxane and ionic liquids: [C_4_C_1_im][MeSO_4_], [C_4_C_1_im][OTf], [C_4_C_1_im][PF_6_] and [C_4_C_1_im][OTf]/[C_4_C_1_im][PF_6_] (1/1 *v*/*v*) binary system were carried out in closed glass vials. Dioxane was dehydrated over 4 Å molecular sieves and ILs were dried at 80 °C under vacuum for 4 h (Rotavapor R-200, Büchi, Rungis, France) prior to use. Both initial water content (<0.03% *w*/*w*) and initial water activity (<0.25) of each reaction medium were determined by the Karl Fischer coulometry method (831 KF Coulometer, Metrohm, Courtaboeuf, France) and a thermoconstanter (LabTouch Aw, Novasina, Lachen, Switzerland), respectively. In a typical reaction in single-solvent system, Kraft lignin (1% *w*/*v*) and ethyl oleate (0.24 M) were added to dioxane or single ILs (0.5 mL) and then incubated for 24 h at 55 °C under vigorous stirring to favor a suitable homogenization of the reaction medium. In the binary systems, lignin and ethyl oleate were separately incubated in [C_4_C_1_im][OTf] and in [C_4_C_1_im][PF_6_], respectively. After 24 h at 55 °C, the two distinct IL solutions were combined in a single stirred flask.

The final concentrations of lignin and ethyl oleate were 1% *w*/*v* and 0.24 M (weight ratio of 1:7.4), respectively. All reactions were started by the addition of 10 g·L^−1^ of immobilized lipase preparation in the medium.

For reactions catalyzed by [C_4_C_1_im][PF_6_]-coated lipases in [C_4_C_1_im][OTf] as single solvent, the coating was prepared according to Mutschler *et al.* [56]. Each immobilized lipase (5 mg) was added to [C_4_C_1_im][PF_6_] (5.15 mg) and acetonitrile (100 µL). The mixture was placed for 20 min at 35 °C under vacuum (Savant SPD 121P SpeedVac concentrator, Thermo Scientific, Villebon-sur-Yvette, France) to evaporate the acetonitrile and then kept for 24 h in a desiccator before use.

Control experiments without enzyme were also carried out in these distinct media. After 48 h of reaction, immobilized enzyme was removed by filtration. For reactions in dioxane, solvent was evaporated under vacuum (Savant SPD 121P SpeedVac concentrator) and residual acyl donors were solubilized in methanol before HPLC analyses. For reactions in ILs, unreacted acyl donors were extracted with hexane (3 *v*/*v*). Hexane was evaporated and the residual acyl donors was solubilized in methanol, and then quantified by HPLC. Partition coefficient of acyl donors in each IL/hexane biphasic system were determined according to previous studies [20,33] then used to correct the concentration values. Each reaction was repeated in triplicate.

### 3.4. Lignin Recovery after Enzymatic Esterification

After enzymatic esterification in dioxane, the solvent was totally evaporated under vacuum. The resulted residue was collected by vacuum filtration and thoroughly washed with hexane to eliminate residual unreacted acyl donor on lignin and then dried for 4 h at 100 °C. The following extraction procedures of lignin from ILs were carried out by water precipitation according to previous studies [50,67]. In addition, two washing steps were successively performed under ultrasound with hexane and then acetonitrile to eliminate residual unreacted acyl donor and ionic liquids. The solid residue was dried for 4 h at 100 °C.

### 3.5. Quantitative Analysis by HPLC

Ethyl oleate and residual acyl co-product were quantified by HPLC (LC 20 AD, Shimadzu, Marne-la-Vallée, France) equipped with an UV detector at 214 nm and a light-scattering low temperature evaporative detector (ELSD-LT II, Shimadzu). The detailed method was described in previous studies [20,33].

Transesterification yield (Y) was determined applying the following equation and expressed as mean values with standard deviations (±):

(1) $$Y\;\left(\%\right)=\frac{[\text{ethyl oleate}]\text{to}–\left(\left[\text{ethyl oleate}\right]\text{t}=48\text{ h}+\left[\text{residual acyl co}-\text{product}\right]\text{t}=48\;h*\right)}{\left[\text{ethyl oleate}\right]\text{to}}\times 100$$

* if detected in the reaction medium.

### 3.6. Structural Characterization

Esterified Kraft lignin was characterized by infrared spectrometry using a FTIR-8400S instrument (Shimadzu) equipped with a universal ATR sampling accessory with diamond crystal. Samples were analyzed between 4000 and 600 cm^−1^ using 128 scans with a resolution of 4 cm^−1^. All spectra were normalized at 1510 cm^−1^, the band assigned to aromatic rings vibration [57,68]. ^1^H-NMR spectra were acquired on an Avance III HD 500 MHz spectrometer (Bruker, Wissenbourg, France) equipped with BBI 5 mm probe operating at 500.0800 MHz. Lignin samples were solubilized in DMSO-*d*_6_. ^1^H-NMR spectra consisting of 1024 scans of 65 k points with spectral width of 7002.801 Hz were collected with acquisition time of 4.7 s and a relaxation delay of 1 s at 300 K. The residual methyl protons signal of DMSO-*d*_6_ at 2.50 ppm was used as reference.

### 3.7. Thermal Analyses

Differential scanning calorimetric analyses (DSC) were carried out on a DSC 204 F1 heat flux differential calorimeter (Netzsch, Selb, Germany) at a constant heating rate of 10 °C·min^−1^, from room temperature up to 160 °C (1st heating), then cooling down to room temperature and finally up to 200 °C (2nd heating), under a constant argon flow of 200 mL·min^−1^.

Thermal Gravimetric Analyses (TGA) were performed on a Simultaneous Thermal Analyzer STA 449 C Jupiter Unit (Netzsch), at a heating rate of 10 °C·min^−1^ under a constant argon flow of 50 mL·min^−1^ and from room temperature to 800 °C. Values of isothermal drift and sensitivity are 0.6 μg·h^−1^ and 0.1 μg respectively. The TGA apparatus is coupled with a Quadrupole QMS 403 Aeolos mass spectrometer MS (Detector SEV/Sekundär Elektronen Vervielfacher (Channeltron) (Netzsch), stainless steel capillary, counting time 20 ms per *m*/*z* with a resting time of 1 s, scanning width 1/51 amu).

### 3.8. Scanning Electron Microscopy

Lignin samples were characterized with an ESEM QUANTA 200 FEG environmental high-resolution electron scanning microscope (FEI Company, Hillsboro, OR, USA). Analyses were performed in low-vacuum mode (under partial vacuum pressure of water) without sample preparation step. The mode of observation was in a secondary electron mode to enable a contrast in topography with a chemical component.

## 4. Conclusions

Lipase-catalyzed transesterification of Kraft lignin with ethyl oleate in hydrophilic/hydrophobic IL media succeeded for the first time. CALB provided superior efficiency in both binary and IL-coated systems. Concerning PCL, the IL-coating allowed significant yield improvements. The possibility to minimize the consumption of hydrophobic IL in maintaining or improving performances was demonstrated in using it as coating agent for lipase rather than as a solvent. Transesterification was proved by spectroscopy and thermal analyses. Significant transesterification yields were obtained by medium engineering with a reaction system combining solubilization of lignin at moderate temperature, increasing the nucleophilicity of its hydroxyl groups while preserving enzymatic activity. In addition, the synthesized bio-sourced material exhibits new interesting textural and thermal properties different from those of the original Kraft lignin, along with a softer and more porous structure. This mild enzymatic strategy in non-conventional media constitutes a promising alternative option for lignin valorization.

## Supplementary Materials

Supplementary materials can be accessed at: http://www.mdpi.com/1420-3049/20/09/16334/s1.
