# Supplementary material for: Enzymatic Transesterification of Kraft Lignin with Long Acyl Chains in Ionic Liquids
Source: Molecules. 2015 Sep 9;20(9):16334–53. doi: 10.3390/molecules200916334 (PMC6332217; doi:10.3390/molecules200916334)
Supplement: Supplementary file 1 [file molecules-20-16334-s001.pdf]

Supplementary Information

Table S1. FT-IR absorbance characteristics of original Kraft lignin between 4000 and 600 cm^{-1} according to literature data [1–3].

**Bands Origin**	**Wavenumber (cm^{-1})**
Phenolic and aliphatic hydroxyl groups	3510
C-H stretching in aromatic methoxyl and methyl plus methylene groups	2936
	2843
Aromatic rings vibrations	1593
	1510
C-H asymmetric deformation	1454
C-H deformation and aromatic ring vibration	1425
O-H phenolic and aliphatic C-H in methyl groups	1369
vibration of G units with C=O stretching	1265
C-C, C-O and C=O stretching	1213
C-H in plane deformation of G units	1142
C-H in plane deformation plus C-O stretching	1030

Reference

1. Schorr, D.; Diouf, P.N.; Stevanovic, T. Evaluation of industrial lignins for biocomposites production. *Ind. Crop. Prod*. **2014**, *52*, 65–73.
2. Chu, S.; Subrahmanyam, A.V.; Huber, G.W. The pyrolysis chemistry of a β-*O*-4 type oligomeric lignin model compound. *Green Chem*. **2013**, *15*, 125–136.
3. Guigo, N.; Mija, A.; Vincent, L.; Sbirrazzuoli, N. Molecular mobility and relaxation process of isolated lignin studied by multifrequency calorimetric experiments.*Phys. Chem. Chem. Phys*. **2009**, *11*, 1227–1236.
